# A systematic review of smartphone applications and devices for obstructive sleep apnea

**DOI:** 10.1016/j.bjorl.2022.01.004

**Published:** 2022-02-04

**Authors:** Peter M. Baptista, Fabricio Martin, Harry Ross, Carlos O’Connor Reina, Guillermo Plaza, Manuele Casale

**Affiliations:** a Clínica Universidad de Navarra, Otorhinolaryngology Department, Pamplona, Spain; b Hospital de Trauma y Emergencias Dr. Federico Abete, Otorhinolaryngology Department, Malvinas Argentinas, Buenos Aires, Argentina; c 3405 Penrose place, Suite 201, Boulder, CO, United States; d Hospital Quironsalud Marbella, Otorhinolaryngology Department, Marbella, Spain; e Universidad Rey Juan Carlos, Hospital Sanitas La Zarzuela, Hospital Universitario de Fuenlabrada, Otorhinolaryngology Department, Madrid, Spain; f Campus Bio-Medico University, Otorhinolaryngology Department, Roma, Italy

**Keywords:** Sleep disorders, Apps, Diagnosis of sleep apnea, Sleep apnea treatment

## Abstract

**Objective:**

Sleep is fundamental for both health and wellness. The advent of “on a chip” and “smartphone” technologies have created an explosion of inexpensive, at-home applications and devices specifically addressing sleep health and sleep disordered breathing. Sleep-related smartphone Applications and devices are offering diagnosis, management, and treatment of a variety of sleep disorders, mainly obstructive sleep apnea. New technology requires both a learning curve and a review of reliability. Our objective was to evaluate which app have scientific publications as well as their potential to help in the diagnosis, management, and follow-up of sleep disordered breathing.

**Methods:**

We search for relevant sleep apnea related apps on both the Google Play Store and the Apple App Store. In addition, an exhaustive literature search was carried out in MEDLINE, EMBase, web of science and Scopus for works of apps or devices that have published in the scientific literature and have been used in a clinical setting for diagnosis or treatment of sleep disordered breathing performing a systematic review.

**Results:**

We found 10 smartphone apps that met the inclusion criteria.

**Conclusions:**

The development of these apps and devices has a great future, but today are not as accurate as other traditional options. This new technology offers accessible, inexpensive, and continuous at home data monitoring of obstructive sleep apnea, but still does not count with proper testing and their validation may be unreliable.

## Highlights

• This paper provides a scientific literature review of consumer-direct apps and devices for the diagnosis, monitoring, and treatment of SDB.
• Sleep apnea apps that had published literature or apps that could be used in a clinical setting were included in this systematic review.
• Ten smartphone apps met the inclusion criteria.
• The use of new technologies for diagnosis, monitoring and treatment of SDB hold great promise but remains in the early stages of development.

## Introduction

Sleep is fundamental for both health and wellness. Its poor quality is linked to increased risk of mood disorders, erectile dysfunction, cardiovascular diseases, diabetes, obesity, and mortality.^1^, ^2^ Sleep disorders span all economic and racial spectrums globally which increases the research and development of sleep health technology with a corresponding surge in new mobile electronic diagnostic and therapeutic options.

Sleep Disordered Breathing (SDB) are varied with many diverse etiologies, each requiring expertise and thoughtful evaluation for proper diagnosis. Until recently, testing could only be performed in dedicated sleep labs at significant time and cost. A gold standard sleep lab study requires patients to spend 12–24 h hard-wired to sensors in a closed room under constant observation by specialist technicians.^3^, ^4^, ^5^, ^6^ Expensive, labor-intensive, and with very low patient turnover, sleep labs are unable to keep up with demand, often booked out six months or more.

Patients, especially those with mild disease, may be reluctant to devote the time and expense needed for this level of evaluation. Facility backlog and patient inconvenience, combined with the advent of “on a chip” and “smartphone” technologies, have created an explosion of inexpensive, at-home applications and devices specifically addressing sleep health.^7^ Patients avidly embrace alternatives to traditional sleep labs, and physicians must stay up to date and preferably lead in this new era.^8^

Dozens/Hundreds^9^, ^10^ of sleep-related smartphone Applications (Apps) are easily downloaded from internet marketplaces offering diagnosis, management, and treatment of a variety of sleep disorders, mainly. These span from simple white-noise generators to sophisticated trackers of sleep time and quality utilizing phones internal motion sensors and gravimeters. Linked devices, such as wearable sleep trackers, provide additional home-based options offering even further data collection and disease management.^11^ These have not been chosen in this paper except does that are linked to CPAP use.

New technology requires both a learning curve and a review of reliability, quality and validity.^12^, ^13^, ^14^, ^15^

Health care providers and healthcare organizations are needed to help evaluate and provide guidance on utility, accuracy, and place in the treatment spectrum.

## Methods

This paper provides a scientific literature review of consumer-direct apps and devices for the diagnosis, monitoring, and treatment of SDB.

The authors searched for relevant apps pertaining to sleep apnea in both the Google Play store and Apple App Store. The keywords used were: “snoring”, “sleep apnea”, “Obstructive Sleep Apnea Syndrome” (OSAS) for the category of sleep medicine. A systematic review from PubMed, Scopus and mobile device app marketplaces (Apple Store and Google Play Store) was performed.

A comprehensive literature search was conducted in MEDLINE (OVID), EMBase, ISI web of science and Scopus for English and Spanish language citations published from January 1, 2008 (considering that the first mobile phone app store was started in mid-2008) to September 1, 2020. A Boolean search strategy using key words related to “mobile health applications” (e.g., mHealth apps OR mobile health applications OR mobile medical applications OR medical smartphone applications) and keywords “sleep apnea”, “sleep tracker devices’, “sleep monitoring”, “snoring”, “obstructive sleep apnea”, “sleep related breathing disorders” “myofunctional therapy for OSA apps” “CPAP treatment apps”. Please see detailed including a list of selected manuscripts (Table 1).

**Table 1:** Selected papers of the systematic research.

Article	Authors	Pubmed/Scopus
Classifying obstructive sleep apnea using smartphones.	Al-Mardini M, Aloul F, Sagahyroon A, Al-Husseini L.	Pubmed
A review of current sleep screening applications for smartphones.	Behar J, Roebuck A, Domingos JS, Gederi E CG	Pubmed
SleepAp: An automated obstructive sleep apnoea screening application for smartphones.	Behar, J., Roebuck, A., Shahid, M., Daly, J., Hallack, A., Palmius, N.	Pubmed
Is there a clinical role for smartphone sleep apps? comparison of sleep cycle detection by a smartphone application to polysomnography.	Bhat S, Ferraris A, Divya Gupta, et al.	Pubmed
Sleep devices: wearables and nearables, informational and interventional, consumer and clinical.	Bianchi, M.T.	Scopus
Smartphone apps for snoring.	Camacho M, Robertson M, Abdullatif J, Certal V, Kram YA, Ruoff CM, Brietzke SE, Capasso R	Pubmed
Unobtrusive sleep monitoring using smartphones.	Chen, Z., Lin, M., Chen, F., Lane, N.D., Cardone, G., Wang, R., Li, T., Chen, Y., Choudhury, T., Campbell, A.T.	Scopus
Smartphone applications to support sleep self-management: Review and evaluation.	Choi, Y.K., Demiris, G., Lin, S.-Y., Iribarren, S.J., Landis, C.A., Thompson, H.J., McCurry, S.M., Heitkemper, M.M., Ward, T.M.	Scopus
Can smartphone apps be used to screen for obstructive sleep apnea.	Duggal C, Pang KP, Rotenberg BW.	Pubmed
Monitoring healthy and disturbed sleep through smartphone applications: a review of experimental evidence.	Fino, E., Mazzetti, M.	Scopus
Smart sleep tracking through the phone: Findings from a polysomnography study testing the reliability of four sleep applications.	Fino, E., Plazzi, G., Filardi, M., Marzocchi, M., Pizza, F., Vandi, S., Mazzetti, M.	Scopus
Current and future roles of consumer sleep technologies in sleep medicine.	Goldstein C.	Pubmed
Smartphone-based delivery of oropharyngeal exercises for treatment of snoring: a randomized controlled trial.	Goswami U, Black A, Krohn B, Meyers W, Iber C.	Pubmed
Treatment of supine position-related obstructive sleep apnea with smartphone applications.	Haas D, Birk R, Maurer JT, Hörmann K, Stuck BA, Sommer JU.	Pubmed
Sleep tracking apps' design choices: A review	Hosszu, A., Rosner, D., Flaherty, M.	Scopus
Monitoring progress and adherence with positive airway pressure therapy for obstructive sleep apnea the roles of telemedicine and mobile health applications.	Hwang, D.	Pubmed
Sleep assessment devices: types, market analysis, and a critical view on accuracy and validation.	Ibáñez, V., Silva, J., Navarro, E., Cauli, O.	Scopus
A New mHealth application to support treatment of sleep apnoea patients.	Isetta V, Torres M, González K, Ruiz C, Dalmases M, Embid C, Navajas D, Farré R, Montserrat JM.	Pubmed
Prediction of obstructive sleep apnea based on respiratory sounds recorded between sleep onset and sleep offset.	Kim, J.-W., Kim, T., Shin, J., Choe, G., Lim, H.J., Rhee, C.-S., Lee, K., Cho, S.-W.	Scopus
Consumer sleep technologies: A review of the landscape.	Ko, P.-R.T., Kientz, J.A., Choe, E.K., Kay, M., Landis, C.A., Watson, N.F.	Scopus
Sleep apps: What role do they play in clinical medicine?	Lorenz, C.P., Williams, A.J.	Scopus
Apps and fitness trackers that measure sleep: Are they useful?	Mansukhani, M., Kolla, B.	Pubmed
Monitoring sound to quantify snoring and sleep apnea severity using a smartphone: proof of concept.	Nakano H, Hirayama K, Sadamitsu Y, Toshimitsu A, Fujita H, Shin S, Tanigawa T.	Pubmed
Contactless sleep apnea detection on smartphones.	Nandakumar, R., Gollakota, S., Watson, N.	Pubmed
New mHealth application software based on myofunctional therapy applied to sleep-disordered breathing in non-compliant subjects.	O’Connor Reina C, Plaza G, Ignacio-Garcia JM, Baptista Jardin P, Garcia-Iriarte MT, Casado-Morente JC, De Vicente Gonzalez E, Rodriguez-Reina A.	Pubmed
Myofunctional therapy app for severe apnea-hypopnea sleep obstructive syndrome: A pilot randomized controlled trial.	O’Connor-Reina C, Ignacio-Garcia JM, Rodriguez-Ruiz E, Morillo Dominguez MDC, Ignacio Barrios V, Baptista Jardin P, Casado Morente JC, Garcia Iriarte MT, Plaza G.	Pubmed
Technologic advances in the assessment and management of obstructive sleep apnoea beyond the apnoea-hypopnoea index: A narrative review.	O’Mahony, A., Garvey, J., McNicholas, W.	Pubmed
Overview of smartphone applications for sleep analysis.	Ong, A., Gillespie, M.	Pubmed
Accuracy of a smartphone application in estimating sleep in children.	Patel P, Kim JY, Brooks LJ	Pubmed
Alternative algorithms and devices in sleep apnoea diagnosis: what we know and what we expect.	Penzel T, Fietze I, Glos M.	Pubmed
An activity tracker and its accompanying app as a motivator for increased exercise and better sleeping habits for youths in need of social care: Field Study. JMIR Mhealth Uhealth.	Rönkkö K.	Scopus
Digital health and sleep-disordered breathing: A systematic review and meta-analysis.	Rosa T, Bellardi K, Viana A, Ma Y, Capasso R.	Pubmed
Digital health and sleep-disordered breathing: A systematic review and meta-analysis.	Rosa, T., Bellardi, K., Viana, A., Ma, Y., Capasso, R.	Scopus
Internet of things for sleep tracking: wearables vs. Nonwearables.	Sadek, I., Demarasse, A., Mokhtari, M.	Scopus
A pilot study of a novel smartphone application for the estimation of sleep onset.	Scott H, Lack L, Lovato N	Pubmed
Apps in sleep medicine.	Stippig A, Hübers U, Emerich M	Pubmed
Validation of contact-free sleep monitoring device with comparison to polysomnography.	Tal, A., Shinar, Z., Shaki, D., Codish, S., Goldbart, A.	Scopus
Screening for obstructive sleep apnea with novel hybrid acoustic smartphone app technology.	Tiron R, Lyon G, Kilroy H, Osman A, Kelly N, O’Mahony N, Lopes C, Coffey S, McMahon S, Wren M, Conway K, Fox N, Costello J, Shouldice R, Lederer K, Fietze I, Penzel T.	Pubmed
A viable snore detection system: Hardware and software implementations.	Tuncer, A.T., Bilgen, M.	Scopus
Sleep parameter assessment accuracy of a consumer home sleep monitoring ballistocardiograph beddit sleep tracker: A validation study.	Tuominen, J., Peltola, K., Saaresranta, T., Valli, K.	Scopus
Sleep apps and the quantified self: Blessing or curse?	Van den Bulck, J.	Scopus
A global quantification of “normal” sleep schedules using smartphone data.	Walch, O., Cochran, A., Forger, D.	Pubmed
Effect of a patient engagement tool on positive airway pressure adherence: analysis of a German healthcare provider database.	Woehrle H, Arzt M, Graml A, Fietze I, Young P, Teschler H, Ficker JH	Pubmed
Quality analysis of smart phone sleep apps in China: Can apps be used to conveniently screen for obstructive sleep apnea at home?	Xu, Z.-F., Luo, X., Shi, J., Lai, Y	Scopus

**Figure 1:**
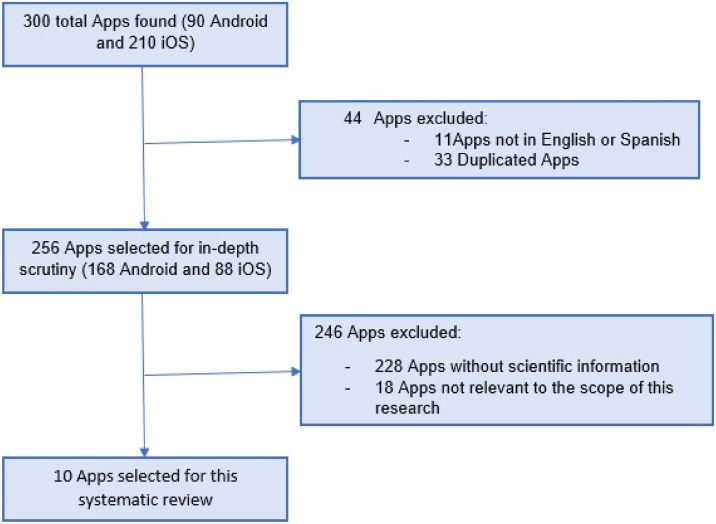
Summary of Apps evaluating for SBD.

**Figure 2:**
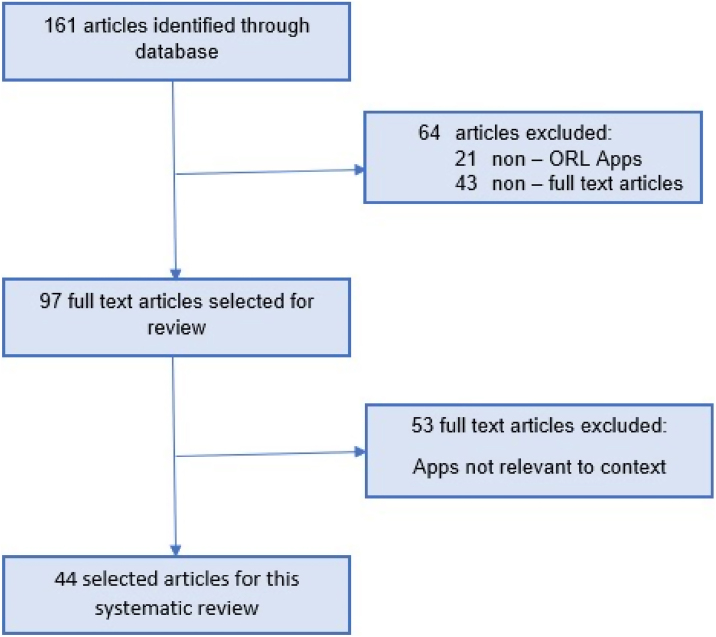
Summary of scientific publications evaluating performance of Apps and devices for SBD.

Sleep apnea apps that had published literature or apps that could be used in a clinical setting were included in this systematic review, while apps not relevant to the scope of this research and/or duplicates, educational or divulgation apps, apps promoting a business or individual, apps requiring specific hardware that had to be purchased separately, and apps in non-English languages were excluded. Furthermore, we searched the National Library of Medicine through PubMed for articles published from inception to a complete review of all apps that were related to the terms was done in Apple Store and Google’s store. Please see Prisma method of selection (Figure 1, Figure 2).

### Data abstraction and study quality assessment

Authors PB, FM, and HR independently performed a search of the literature and screened titles and abstracts and downloaded the articles for inclusion. The decision to include the articles was made by consensus, and if necessary, the final decision was made by author PB. Data collected included: polysomnographic data, (AHI, lowest oxygen saturation), snoring, sleepiness data, usage, type of app (diagnosis, therapy), data type (obtained directly from app through sensors or questionary). If data were missing from the articles, then the corresponding author was contacted to obtain the data.

The National Institute for Health and Clinical Excellence (NICE) quality assessment tool was used to evaluate the quality of the included studies. The instrument consists of eight items that are assessed for each individual study. By doing so, the risk of bias assessment was minimized.

## Results

We found a total of 300 apps in Android and Apple store. Of those 44 were excluded for not being in English or were duplicates. A total of 256 were selected in depth for scrutiny (Fig. 1). Only 10 smartphone apps that met the inclusion criteria of having solid scientific data with publications (Table 2).

**Table 2:** Apps included in this research.

App	Aim	Platform	Cost (U$D)
Sleep time	Diagnostic	iOS/Android	1.99
Sleep Cycle	Diagnostic	iOS/Android	1.99
Snore Lab	Diagnostic	iOS/Android	0.99
Sleep on Q	Diagnostic	iOS	0.99
Resmed My Air	Measure treatment, advice, reminder	iOS/Android	Free
APPnea-Q	Advice for Cpap treatment	iOS/Android	Free
Airway Gym	Myo-facial treatment	iOS/Android	Free
Snoretech	Snore tracker & Myofacial terapy	Android	4.99‒5.30
Apnea Sleep Position Trainer	Positional obstructive sleep apnea therapy apps	iOS/Android	Free
SomnoPose ‒ Sleep Position Monitor	Positional obstructive sleep apnea therapy apps	iOS	9.49

### Stand-alone smart phone apps for sleep monitoring

#### Sleep time

Provides users with a graph detailing level of wakefulness and light/deep sleep. Features include a “smart” alarm clock, engineered to wake the user when the app senses a period of “light sleep”, designed to produce a more pleasant awakening experience.

Clinical Research: Bhat et al.^16^ compared the Sleep Time app to PSG in adults and found a poor correlation between the app and PSG in terms of sleep efficiency, light sleep, and deep sleep.

No correlation was found between the app and PSG sleep latency. App overestimated sleep latency by 15.6 min, indicating a deficiency in terms of detecting wakefulness. No evidence was found to support the claim of app consistently awakened subjects during light sleep.

#### Sleep cycle

Accelerometer-based app designed to ease awakening during optimal light sleep periods.

Clinical Research: Patel et al.^17^ examined the app by comparing its sleep analysis with PSG in a clinical population of 25 children (age 2–14) undergoing overnight PSG for clinical suspicion of OSA. No significant correlation was found between total sleep time and sleep latency compared to PSG. Only sleep latency from the PSG and latency to deep sleep were found to have a significant relationship. The authors concluded that Sleep Cycle App is not yet accurate enough to be used for clinical purposes.

#### Sleep on Q

Behavioral training response to auditory stimuli estimates sleep onset. It gauges sleep onset if the user fails to respond to a series of audible tones.

Clinical Research: Scott et al.^6^ found high correspondence between the app and PSG sleep onset. App tended to overestimate sleep latency. The authors highlight the potential relevance of use for facilitating power naps in the home environment.

#### SnoreLab

Monitors and provides feedback on auditory snore activity.

Clinical Research: Stippig et al.^18^ tested the ability to distinguish between snoring events and other background noise. Results did not correspond with concurrent validated ApneaLink Plus screening device, which led authors to conclude reliability and accuracy are insufficient to replace common diagnostic standards.

### Smart phone apps for adjunct monitoring CPAP use

CPAP devices work by generating positive airway pressure at the pharynx level, preventing airway collapse, eliminating snoring, hypopnea, and obstruction events. CPAP efficacy and indications are well known, but compliance can be poor.^19^ Multiple efforts are made to improve adherence.

#### ResMed My Air™

An engagement tool that allows patients to track nightly sleep data and through interactive coaching empowers patients to stay engaged and compliant with long-term therapy.

Clinical Research: Woehrle et al.^20^ analyzed a large cohort of patients managed under routine clinical practice conditions. This tool’s addition was associated with significant compliance improvement in first-time users receiving PAP therapy, irrespective of the interface used. Increases were seen in both nightly hours of device use and the number of days of device usage. Compared to proactive care (telemonitoring alone), patients utilizing “engagement management” approach demonstrated a significant reduction in air leakage.

#### Appnea-Questions (Appnea-Q)

Aims to improve CPAP adherence by a series of text message questions. Patients are asked to answer three daily yes/no questions about OSA treatment concerning CPAP use, physical activity, dietary habits, and a weekly input of body weight. Users are provided concise recommendations about CPAP use and a healthy lifestyle. Weekly and global summaries of questionnaire answers are available in a graphical format.

Clinical Research: Isetta et al.^21^ evaluated 60 naive CPAP patients. Regular users of Appnea-Q had significantly higher CPAP compliance. Satisfaction levels were high for most users.

### Apps for OSA treatment

#### Airway gym

Orofacial Myofunctional Therapy (OMT), or oropharyngeal exercises have been recently used with success, as a treatment for reducing OSA severity. It is proposed that by strengthening oropharyngeal muscles through daily exercise, upper airway collapse is avoided. OMT is useful for the treatment of adult patients with mild and moderate OSA and with primary snoring,^22^, ^23^, ^24^ and of children with residual apnea.^25^, ^26^ The main problem of this therapy is the lack of adherence of up to 10% in most of the studies reported.^27^, ^28^

The app provides a permanent record of feedback and accuracy of the exercises carried out while interacting with the screen. In a recent study, O’Connor et al.^29^ also showed in 15 of 20 (75%) patients using the device an improvement their AHI from 25.78 ± 12.6 to 14.1 ± 7.7 (*p* = 0.002), ESS (Epworth sleepiness score) from 18.2 ± 1.98 to 14.2 ± 7.7 (*p* = 0.002) and SatO_2_MIN from 84.87 ± 7.02 to 89.27 ± 3.77 (*p* = 0.0189) after performing daily app exercises of the app for 3-months compared with a control group.

Recently it was published a randomized trial^30^ with this app in severe OSAHS patients where the AHI decreased by 53.4% from 44.7 (33.8‒55.6) to 20.88 (14.02‒27.7) events/hour (*p* < 0.001). The oxygen desaturation index decreased by 46.5% from 36.31 (27.19‒43.43) to 19.4 (12.9‒25.98) events/hour (*p* = 0.003). The Epworth Sleepiness Scale score decreased from 10.33 (8.71‒12.24) to 5.37 (3.45‒7.28) in the app group (*p* < 0.001), but the Pittsburgh Sleep Quality Index did not change significantly. They got an adherence of 90% in the intervention group.

#### Snoretech

Users perform 15-minutes of daily voice-activated gameplay to improve snoring and sleep quality. Users articulate specific phonemes to achieve voice-controlled on-screen objectives. Exercises are focused on improving endurance, strength, and coordination of upper airway muscles by repeatedly moving tongue base forward and backward.

Clinical Research: Randomized controlled trial with snorer patients shows significant reduction in snoring and ESS after 8-weeks.^31^

### Positional obstructive sleep apnea therapy apps

Positional therapy is defined as any technique used to avoid problematic sleeping positions, which can cause positional obstructive sleep apnea.^32^ A couple of sleep positions are available and used for the treatment of Positional Obstructive Sleep Apnea (POSA): Apnea Sleep Position Trainer (Con4m, Hoofddorp, Netherlands). It is provided in both the iOS and the Android software platform. It does not have a data memory and only differentiates between prone, back and side positions. Only the night immediately preceding can be displayed; SomnoPose ‒ Sleep Position Monitor (Proximal Box Software, Eagan/MN, USA). In addition to the general functions of position detection and vibration alarm, this app offers a detailed history of the position during the night and a memory of the nights that have occurred recorded retrospectively.

Both use the position sensors used in smartphones and have a vibration alarm. The smartphone needs to be attached to the chest to recognize SP, which then triggers a vibration alarm. This is intended to encourage the patient to change position and the vibration stops as soon as SP is left.

In a study described in the University of Manheim^33^ including 33 patients who ﬁnished the study, both smartphone apps showed the capability to prevent POSA patients and can potentially oﬀer a cost-eﬀective option in the treatment of POSA. The overall AHI was reduced from 14.5 ± 9.0 to 9.5 ± 12.6 and the time in supine position decreased signiﬁcantly from 71.1 ± 50.5 to 25.4 ± 65.0 min. Compliance after 6-months was 79.2%.

## Discussion

Daily internet use is a regular part of most people's lives with personal health, disease, and diagnosis searches rapidly increasing. Patients presenting for consult often arrive with information gleaned from the web, requesting doctors to now opine not just on basic health but new medical apps and devices. This poses a challenge for physicians who, in addition to disease, diagnosis, and management, must also now be fluent and up to date in unregulated direct to consumer electronic products.

The ubiquitous smartphone and their ever-increasing associated number of apps and linked devices has created a great promise for at home, patient-centric health. Direct to consumer diagnosis, monitoring, and treatment of SDB theoretically offers an inexpensive convenience for patients eager to engage with their own medical workup. Useful or merely creating an inaccurate distraction, unrivaled accessibility, and ever-expanding abilities of the all-pervasive smartphone is creating a patient-driven shift in diagnosis and treatment of SDB from clinic to home.

Smartphone apps are being developed at dizzying speed with new ones seemingly available each day. As with many consumer-oriented products, quality can vary greatly. In health care, this can and should be of great concern.^34^ While some work well and deliver on stated function, others may be inaccurate or too immature in their current technological development cycle for reliable use.

Sleep clinicians need to have an approach to the patient who recognizes the limitations of Consumer Sleep Technology (CST). The American Academy of Sleep Medicine published in 2018 an important paper to try to guide physicians on how to approach the patient, that has great interest in CST, recognizing the potential benefits of these, however, given the lack of scientific validation most CSTs cannot be utilized for the diagnosis and/or treatment of sleep disorders at this time. But CSTs may be utilized to enhance the patient-clinician interaction when presented in the context of an appropriate clinical evaluation.^8^

Peer-reviewed publications that summarize the performance of currently available CSTs compared with PSG remain limited.^35^, ^36^, ^37^ The importance of these apps come to surface especially during this hard year of the COVID-19 pandemic where there are restricted healthcare resources and sleep medicine services are advised to reduce in-house services, and to provide medical care by remote monitoring using phone, video calls and telemedicine solutions.^38^

Recently, we have seen the development of more complex technology with underpinning Software Development Kit (SDK), which utilizes advanced Digital Signal Processing (DSP) technology and Artificial Intelligence (AI) algorithms to identify detailed sleep stages, respiration rate, snoring, and OSA patterns seems to allow a higher degree of sensitivity and specificity.^39^

### Certain APP trends were noted in this review

For snoring and OSA detection devices, diagnostic sensitivity and specificity appeared acceptable only for moderate and severe disease,^18^ but delivered a poor performance with mild disease.

Questionnaire-based sleep applications increased adherence to self-monitoring and self-report rates of subjects, possibly due to ease of use and constant availability.^40^

The use of apps can notably improve compliance and adherence to exercise and training regimes.

Xu et al. in a recent paper showed that when apps are connected to other devices, these seemed to be more scientific and reasonable. People can use these apps with accessory devices to monitor their sleep conveniently.^41^

As motivators for compliance apps appear to work well and much better then linked devices. The current literature review suggests that despite a promise, many 1^st^ and 2^nd^ generation devices perform poorly in reporting absolute parameters or staging sleep and sleep-wake cycles. Despite benefit claims, few devices have been validated against PSG, and accuracy tends to drop off with low sleep efficiency when most needed.

It is unclear if this represents the industry as a whole or is simply a reflection of selection bias in reviewing only those “popular” apps/devices.

In performing this review, the authors also noted several recurrent issues. Acceptable level 1 data is scarce. Many of the cited studies were run on healthy volunteers with neither SDB nor formal sleep lab controls. As these are non-validated and self-reported, it is not easy to assess results accurately. Without a confirmed and standardized cohort, claims for utility are not possible.

Most app are exempt from formal regulatory review/approval and skirt this issue by not making strict medical claims. Language such as “assists in” replaces “diagnosis” or “treatment of”. Strict European device regulations taking effect in May 2020 attempt to address this issue, but regulation of medical apps lags significantly. The Android and Apple stores appreciate this grey zone as they are also cautious not to imply direct disease diagnosis or treatment but instead allow ambiguous goals such as “treatment or assessment” of respiratory disturbances in the case of snoring and sleep apnea.

Apps may be popular related to their functionality and Usability. Xu et al.^41^ pointed out that people prefer multifunctional apps that could provide information about sleep, could play sleep-inducing music and could be used as a smart alarm clock to help people wake up at the best time. Apps that also provide the capacity to consult with a doctor tend to be more popular in China.^42^ There is not, however, a correlation with the scientific domain that suggests that apps with multiple functions were not necessarily better than other apps.

As limitations to our study, while 256 apps were evaluated in this study, it is possible that some apps that met the inclusion criteria were missed. Some apps may be limited exclusively to certain countries not been able to download in others.

An ideal app that can monitor sleep and screen for OSA should be designed by a collaboration between app designers and doctors. Studies have indicated increasing acceptance for remote sleep monitoring and screening for OSA.^43^, ^44^

## Conclusion

Consumer-targeted apps that support sleep self-management can raise awareness and promote healthy sleep habits. Smartphone technology provides potentially beneficial opportunities for increased patient interest and constructive treatment engagement. Increased patient enthusiasm encourages productive engagement and collaboration with clinicians in successfully developing and implementing treatment goals.

The use of new technologies for diagnosis, monitoring and treatment of SDB holds great promise but remains in the early stages of development. Smartphone apps and linked devices offer accessible, inexpensive, and continuous at home data monitoring but without proper testing, and validation may be unreliable. Serious concerns for ethical, security, privacy, and connectivity issues exist within the mHealth realm for Apps with unvalidated “cure “or “treat” claims.^45^ Until such time as validated accuracy is available, smart phone apps/devices for SDB should be used with caution as adjuncts, not replacement for formal sleep studies.

## Compliance with ethical standards

No Funding was received.

This article does not contain any studies with human participants or animals performed by any of the authors.

## Conflicts of interest

The authors declare no conflicts of interest.
